# Interaction Effects of Disruptive Behaviour and Motivation Profiles with Teacher Competence and School Satisfaction in Secondary School Physical Education

**DOI:** 10.3390/ijerph17010114

**Published:** 2019-12-23

**Authors:** Antonio Granero-Gallegos, Manuel Gómez-López, Antonio Baena-Extremera, Marina Martínez-Molina

**Affiliations:** 1 Department of Education, Faculty of Education Sciences, University of Almeria, 04120 Almeria, Spain; agranero@ual.es (A.G.-G.); marinamartinezmolina@gmail.com (M.M.-M.); 2 Health Research Center, University of Almeria, 04120 Almeria, Spain; 3 Department of Physical Activity and Sport, Faculty of Sport Sciences, University of Murcia, Santiago de la Ribera, 30720 Murcia, Spain; 4 International Campus of Excellence “Mare Nostrum”, University of Murcia, 30720 Murcia, Spain; 5 Department of Didactic of Corporal Expression, Faculty of Education Sciences, University of Granada, 18071 Granada, Spain; abaenaextrem@ugr.es

**Keywords:** secondary education, adolescence, satisfaction with school, bored with school, teaching

## Abstract

The objectives of this work were two-fold: Firstly, to identify the profiles of disruptive behaviours and motivation in secondary school physical education students using cluster analysis; and secondly, to analyse the interaction of the profiles with school satisfaction and perceived teaching competence. A group of 758 secondary school students (54.2% female) between the ages of 13 and 18 (*M* = 15.22, *DT* = 1.27) participated in the study by responding to the following scales: The Disruptive Behaviours in Physical Education Questionnaire, The School Satisfaction Scale, The Sport Motivation Scale adapted to Physical Education, and the Evaluation of Teaching Competencies Scale in Physical Education. The cluster analysis established two distinct profiles: High levels of disruptive behaviours and low levels of disruptive behaviours. The results showed that the students with the high disruptive behaviours profile were mostly boys, having low levels of intrinsic motivation and high levels of amotivation and misbehaviour in the classroom. In contrast, those students with the low disruptive behaviours profile were mostly girls, having the highest levels of intrinsic motivation and the lowest levels in all the disruptive behaviours. It was shown that students exhibiting the worse classroom behaviours were more bored in school, while those students with better behaviour perceived greater teaching competence.

## 1. Introduction

The literature shows that the variables that can influence learning, academic performance, school failure, and the personal growth of students are disruptive behaviours in the classroom, teaching competence, student motivation, subjective well-being, and school satisfaction [1,2].

School failure is related to the success or failure of the education system and cannot be addressed today without considering academic problems. Of these, disruptive behaviours in the classroom stand out due to teachers’ concerns about coexistence and the classroom climate [3,4,5,6]. This phenomenon affects most schools, the results of which are manifested in the dysfunction of the educational process [7].

Transgression of the rules is a common behaviour in the evolutionary development of minors during childhood and adolescence, and it is a process that strengthens personality and social positioning. These behaviours are externalized mainly in adolescence during compulsory secondary education (ESO) [8,9], probably due to the important changes that take place during this period and how they develop; for instance, changes in physical appearance and in the psychosocial field. Such psychosocial changes often entail a crisis in the adolescent concerning identity and values, along with an increase in behaviours that affect coexistence both in the family and at school.

Disruptive behaviours interrupt the teaching–learning process and are normally associated with an unfavourable family environment and a mismatch relationship within the school context [10,11]. Jurado and Tejada [12] define disruptive behaviours as those that hinder learning and distort individual relationships, as well as the dynamics of the class, affecting not only the student who provokes them, but also the other students and the teachers who have to endure the consequences [13]. These misbehaviours make it difficult to carry out classroom tasks properly and hinder actions performed in the learning context. Consequently, the disruptive behaviour can be identified by the manifestation of a conflict and/or troubling behaviour contrary to the explicit (or implicit) norms of the educational context [12], jeopardizing the maintenance of an optimal school learning environment and successful teaching [14,15,16].

Because such disruptive behaviour affects the climate of school coexistence, it can be considered as one of the triggers for situations that risk school failure, social maladaptation, and rejection, which we currently witness, above all, in secondary schools [17,18].

Disruptive behaviour typically leads to low academic performance, which influences the student’s risk of failure at school in some way or another. Due to the low academic levels of Spanish students documented in certain international studies (e.g., the PISA report), there has been increasing interest over recent years to study the factors affecting academic/school performance and students’ personal growth [1,19,20].

The scientific literature has shown that there is a relationship between academic performance and satisfaction with school [15], which determines the student’s engagement in his/her schoolwork [21]. Furthermore, recent studies [1] have shown that satisfaction/fun in the subject of physical education (PE) positively predicted the satisfaction/fun experienced at school. This school satisfaction contributes to reducing both the school drop-out rate and disruptive behaviours [22], and can even positively influence the students’ satisfaction with life [2]. Conversely, school dissatisfaction is associated with negative behaviours, such as dropping out of school [23]. It should be noted that the premature abandonment of schooling is related to the manifestation of discipline problems [24]; i.e., those students who tend to present discipline problems tend to drop out of school in a high percentage of cases, especially in ESO, thus undervaluing the role of the school as the main motivation in their life trajectory.

Faced with this problem, the educational possibilities that come from practising sports have been noted in psychosocial development and in the social integration of children and young people [25]. Practising sport provides an appropriate context for enhancing personal values such as self-esteem, perseverance, and the capacity to strive to achieve one’s goals, as well as social values such as belonging to a group, cooperation, respect, and solidarity [26]. In addition, the influence of sport on the adolescent socialization process has been confirmed in various studies, determining that systematic participation makes it possible to reduce antisocial behaviour [27], and that the effects increase when coaches maintain an attitude that promotes prosocial behaviour [28]. Promoting prosocial behaviour is one of the objectives that the educational context must achieve [7].

Other essential elements for creating an adequate climate in the classroom, in this case, in PE classes, are motivation and discipline [29], which help to develop effective teaching–learning processes. The results provided by Moreno et al. [29], in which they followed Self-Determination Theory [30], demonstrated that discipline was positively and significantly related to intrinsic and extrinsic motivation, and negatively and significantly related to lack of motivation. On the other hand, discipline was positively predicted for a motivational, task-oriented climate, and indiscipline was positively predicted for demotivation and an ego-oriented motivational climate. Consequently, the results showed that, to avoid indiscipline or reduce it, teachers must favour the most self-determined forms of motivation, preventing students from falling into demotivation. In this way, students will spend class time doing work rather than being undisciplined, learning to choose appropriate behaviours in the classroom. That is why it is very important that the teacher directs the students through discipline strategies of concern and responsibility in order to achieve self-determined motivation [31,32]. In addition, the literature has shown that students who emphasise intrinsic reasons feel greater satisfaction in PE classes, improve their relationships with others, and value the subject more, attaching greater importance and usefulness to it [33,34]. Such a mindset favours a climate of learning in class.

On the other hand, another possible reason for these disruptive behaviours manifesting themselves is the conflict between the institution’s expectations and those of the teachers and students. This produces personal dissatisfaction, inefficiency in achieving educational objectives, rebelliousness, and indiscipline. Alderman and Green [35] even allude to low-quality relationships between teachers and students as the source of these behavioural problems in the classroom. The teacher is primarily responsible for maintaining control during the class and for detecting and channelling the most common inappropriate behaviours. Various studies have highlighted the importance of the teacher’s role, both in terms of the student’s satisfaction with school and life and in their learning and academic performance [19,36,37,38].

Other research, such as that by Cameron and Lovett [39], linked the teacher’s lack of interest in teaching and the negative impact on pupils with low school engagement and poor academic performance. In addition, the literature reflected that teaching competence in PE classes was a positive predictor of student satisfaction/fun and a negative predictor of boredom [36], whereas teaching incompetence was a positive predictor of inappropriate behaviour among adolescents [37].

Finally, and despite what we already know, the analyses of motivational profiles and disruptive behaviours in class, as well as the interaction of these profiles based on the student’s satisfaction with the school and their perception of teaching competence, have not yet been addressed together, which suggests that these results will be an interesting contribution to the scientific literature. Therefore, considering the existing theoretical knowledge, it was hypothesized that those students with high disruptive behaviours (especially boys) would be associated with a profile of amotivation, boredom with school, and a perception of low teacher competence. The study’s objective was to identify the disruptive behaviours and motivation profiles of secondary school students in PE classes and to evaluate the interaction of these profiles with respect to school satisfaction and the students’ perception of teaching competence.

## 2. Materials and Methods

### 2.1. Participants

The design of this cross-sectional study was observational and descriptive. A nonprobabilistic and convenience sample selection process was employed, based on the subjects that we were able to access. A group of 758 students (45.8% male and 54.2% female) from the Murcia region (Spain) took part. The age range was between 13 and 18 years old (*M* = 15.22, *DT* = 1.27; *M*_boys_ = 15.20, *DT*_boys_ = 1.29; *M*_girls_ = 15.18, *DT*_girls_ = 1.26). The distribution by course year was: 343 (45.3%) from the second year of ESO; 20.1% from the third, 27.2% from the fourth, and 7.5% from the first year of Bachillerato. As PE is a compulsory subject for all students of the first year of Bachillerato, these students were also included in this research. All students are from public schools located in areas of medium socioeconomic level, and no educational center is included in the program of Teaching Compensatory, a program that allocates specific, material, and human resources to guarantee access, permanence, and promotion in the educational system for socially disadvantaged students.

### 2.2. Measurement Instruments

Disruptive Behaviours in Physical Education: The Disruptive Behaviours in Physical Education Questionnaire (CCDEF) by Granero-Gallegos and Baena-Extremera [14] was used. This is the Spanish version of the original Physical Education Classroom Instrument (PECI) by Krech, Kulinna, and Cothran [40]. This version consists of 17 items that measure disruptive behaviours in PE students over five subscales: Aggressive (AGR) (2 items; e.g., “Threatens others/I threaten other classmates”); Low engagement or irresponsibility (LEI) (4 items; e.g., “Lazy/I am lazy in class”); Fails to follow directions (FFD) (4 items; “Not following directions/I do not follow the instructions”); Distracts or disturbs others (DDO) (4 items; e.g., “Leaving the group during an activity/I abandon the group during an activity”); and Poor self-management (PSM) (3 items; e.g., “Makes fun of other students/I make fun of classmates”). A five-point Likert scale ranging from 1 (never) to 5 (always) was used for the responses. The internal consistency indexes (Cronbach’s alpha (α)) were: AGR, α = 0.58, composite reliability = 0.81, Average Variance Extracted (AVE) = 0.54; LEI, α = 0.73, composite reliability = 0.84, AVE = 0.74; FFD, α = 0.77, composite reliability = 0.94, AVE = 0.65; DDO, α = 0.81, composite reliability = 0.92, AVE = 0.80; and PSM, α = 0.84, composite reliability = 0.96, AVE = 0.92. Given the low index achieved by Cronbach’s alpha and that the AGR subscale consists of only two items, this factor was ignored in the analyses performed. The goodness-of-fit indexes of the scale using confirmatory factorial analysis (CFA) were: Chi-squared ratio (χ^2^)/degrees of freedom (gl) = 3.61; incremental fit index (IFI) = 0.96; Tucker–Lewis index (TLI) = 0.95; comparative fit index (CFI) = 0.96; root mean square error of approximation (RMSEA) = 0.06; Standardized Root Mean Square Residual (SRMR) = 0.06.

The Sport Motivation Scale (SMS): We used the Spanish version adapted to PE [41] of the Sport Motivation Scale by Pelletier et al. [42]. It consists of 28 items that measure the different types of motivation established by Self-Determination Theory [41], which suggests the multidimensional explanation of motivation: Amotivation (AMO) (4 items; e.g., “I don’t know anymore; I have the impression of being incapable of succeeding in this sport/I really do not feel capable of physical-sports practice”), extrinsic motivation (EM) (12 items; e.g., “Because people around me think it is important to be in shape”), and intrinsic motivation (IM) (12 items; e.g., “For the pleasure I feel while improving some of my weak points”). Responses were collected on a Likert scale ranging from 1 (totally disagree) to 7 (totally agree). The internal consistency found in this study was: IM, α = 0.91, composite reliability = 0.99, AVE = 0.92; EM, α = 0.91; composite reliability = 0.99, AVE = 0.88; AMO, α = 0.75, composite reliability = 0.85, AVE = 0.58. The goodness-of-fit indexes of the scale using CFA were: χ^2^/gl = 3.02; IFI = 0.96; TLI = 0.97; CFI = 0.98; RMSEA = 0.05; SRMR = 0.04.

School Satisfaction: The Spanish version of the Intrinsic Satisfaction Classroom Questionnaire (ISC) by Castillo, Balaguer, and Duda [43] was used, adapted from the original Intrinsic Satisfaction Classroom Scale by Nicholls, Patashnick, and Nolen [44], Nicholls [45], and Duda and Nicholls [46]. This consists of eight items that measure the degree of satisfaction with school using two subscales measuring satisfaction/fun with school (5 items; e.g., “I normally enjoy learning at school”) and bored with school (3 items; e.g., “At school I usually get bored”). For the responses, a Likert scale was used, ranging from 1 (totally disagree) to 5 (totally agree). The internal consistency indexes were: Satisfaction/fun, α = 0.76; composite reliability = 0.76; AVE = 0.54; boredom, α = 0.70; composite reliability = 0.72; AVE = 0.52. The goodness-of-fit index of the scale using CFA was: χ^2^/gl = 4.79; IFI = 0.96; TLI = 0.93; CFI = 0.96; RMSEA = 0.06; SRMR = 0.04.

Teaching competence: The Spanish version of the Evaluation of Teaching Competencies Scale adapted to Physical Education (ETCS-PE) by Baena-Extremera et al. [36] was used, adapted from the original Evaluation of Teaching Competencies Scale by Catano and Harvey [47]. This consists of eight items measuring students’ perception of teacher effectiveness. A seven-point Likert scale ranging from low (1, 2; e.g., “The teacher refuses to reach consensus with the class when issues are raised, such as the appropriateness of syllabus content or making new arrangements for assigned work”), medium (3, 4, 5; e.g., “The teacher generally deals with student concerns effectively, yet solutions are not always universally accepted, such as omitting material to compensate for lost time”), and high (6, 7; e.g., “The teacher effectively deals with issues that impede learning, such as rearranging lab time due to an oversized class or facilitating group discussions when the material is not clearly understood”). The internal consistency indexes were: α = 0.86, composite reliability = 0.86, AVE = 0.59. The goodness-of-fit indexes of the scale using CFA were: χ^2^/gl = 1.80; IFI = 0.99; TLI = 0.99; CFI = 0.99; RMSEA = 0.03; SRMR = 0.02.

### 2.3. Procedure

Permission to carry out the work was obtained from the competent bodies, whether at the secondary schools or at the university. Parents and adolescents were informed about the protocol and the study’s subject matter. The informed consent of both was an indispensable requirement to participate in the research. The tools measuring the different variables were administered in the classroom by the researchers themselves, without the teacher present. All participants were informed of the study objective, the voluntary and confidential nature of the responses and the data management, as well as their rights as participants thereof, under the Helsinki Declaration [48]. This research was approved by the Research Ethics Committee at the University of Murcia (REF-45-20/01/2016).

### 2.4. Data Analysis

First, the descriptive statistics, the correlation between the subscales, and the internal consistency of each subscale were calculated, as well as the asymmetry and kurtosis, with values ranging between −0.32 and 1.83. The cluster analysis was performed. This is a multivariate technique that seeks to group elements (or variables) to achieve maximum homogeneity in each group and the greatest differences between them. It was intended that the student characteristics found in one group would be similar in some respects and different in others [49]. Based on suggestions by Hair, Anderson, Tathan, and Black [50], two clusters analyses were conducted. The total sample was randomly divided into two groups so that each group was made up of approximately 50% of the sample. Group A consisted of 372 subjects (54.3% girls) and group B consisted of 386 students (54.1% girls). In order to identify the motivational profiles represented in group A, an exploratory hierarchical cluster analysis was performed using the Ward’s method and based on the dendrogram reading, and a solution was selected from the logical results obtained. These results were then verified, obtaining the motivational profiles of group B by cluster analysis using the k-means method. Lastly, a final cluster analysis was performed with the entire sample using the k-means method again. The composition of the profiles was then examined and the subjects identified in each of the profiles formed two different groups (cluster 1 and cluster 2). This acted as an independent variable to analyse the differences in satisfaction with school and teacher competence using multivariate analysis. For these analyses, SPSS v.22.0 (IBM, Chicago, USA) was used, while for the CFA, we used AMOS v.22.0 (IBM, Chicago, USA).

## 3. Results

### 3.1. Descriptive and Correlation Analysis

Table 1 sets out the descriptive values for each of the study variables. For disruptive behaviours, low engagement or irresponsibility presented the highest mean values, while the lowest were for poor self-management. On the motivation scale, the highest mean value corresponded to intrinsic motivation, while the lowest was for amotivation. It is noteworthy that boredom with school had higher values than satisfaction with school. Lastly, the Evaluation of Teaching Competence Scale achieved moderately high values.

The correlations showed a high and significant relationship between the four disruptive behaviour subscales (between 0.59 and 0.79). Furthermore, the correlation between these four subscales and amotivation and boredom with school was significant and positive (between 0.18 and 0.26). On the other hand, disruptive behaviours did not correlate with satisfaction with school, whereas they did in a negative and significant way with teaching competence and intrinsic motivation. Low engagement or irresponsibility and failure to follow directions also correlated negatively and significantly with extrinsic motivation. Teaching competence also showed positive and significant correlations with intrinsic motivation and extrinsic motivation. It did not correlate with amotivation, but did correlate negatively with boredom with school.

### 3.2. Cluster Analysis

The cluster analysis was conducted to study motivational profiles and disruptive behaviours amongst secondary school PE students, adjusting the phases to the procedure designed by Hair et al. [50]. No lost cases were reported in any of the analytical variables, which were standardized using *Z* scores. The students were then grouped together in clusters. First, an exploratory hierarchical cluster analysis was performed to identify the cluster number in group A. Since this analysis is exploratory, it is important to confirm the results with a separate sample; thus, a k-mean cluster analysis (nonhierarchical) was performed on group B. With regard to the multicollinearity between variables, because none of Pearson’s correlation coefficients were >0.90, we decided that there was no multicollinearity problem [50].

The Ward’s method was used in the exploratory analysis of group A (*n* = 372) as this hierarchical procedure minimizes the distance between the subjects within the cluster (it reduces the variance within the group) and avoids forming long chains [49]. The dendrogram suggests tow clusters as the most convenient solution. The first group was named “High disruptive behaviours” (*n* = 43) and included the highest mean values of all disruptive behaviour factors, as well as the highest mean for amotivation. The second profile was labelled “Low disruptive behaviours” (*n* = 328), including students with the lowest mean levels of amotivation and disruptive behaviours and higher mean values for intrinsic motivation and extrinsic motivation (Table 2).

For the analysis of the group B students, a k-means cluster analysis was performed, identifying two profiles. This type of analysis is considered confirmatory, as it requires an a priori provision of the specific number of groupings expected to arise in the sample [51]. The profile findings presented similar characteristics to those found in group A: The “High Disruptive Behaviours Profile” (*n* = 50), characterized by positive Z scores in the four factors of the disruptive behaviours scale and in amotivation, were negative in both intrinsic motivation and extrinsic motivation, although the values were more negative in intrinsic motivation (Table 2); and the “Low Disruptive Behaviours Profile” (*n* = 336), characterized by a negative score for amotivation and disruptive behaviours, while intrinsic motivation and extrinsic motivation scored positively, albeit with values close to zero. Some differences were found regarding the total distribution of students within the groups, highlighting especially the greater representation of students in cluster 2 in both groups.

**Figure 1:**
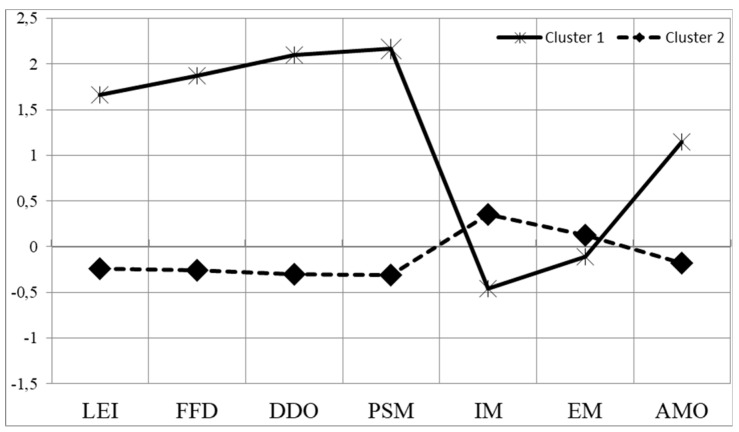
Motivational and disruptive behaviours profiles. Cluster 1: High disruptive behaviours profile; Cluster 2: Low disruptive behaviours profile. Z scores are represented on the vertical axis. On the horizontal axis are the scores of each subscale with the following abbreviations: LEI—Low engagement or irresponsibility; FFD—Fails to follow directions; DDO—Distracts or disturbs others; PSM—Poor self-management; IM—Intrinsic motivation; EM—Extrinsic motivation; AMO—Amotivation.

Finally, the k-means cluster analysis (nonhierarchical) was performed with the total sample, revealing cluster profiles similar to those found in analyses of groups with a random sample (Figure 1; Table 3).

Cluster 1: The first profile grouped a total of 97 students (12.8%) presenting a “High Disruptive Behaviours Profile”. The highest Z scores were found in poor self-management (*Z* = 2.17) and “distracts or disturbs others” (*Z* = 2.10), followed by failure to follow directions, low engagement or irresponsibility, and amotivation. The lowest values corresponded to intrinsic motivation (*Z* = −0.46), followed by extrinsic motivation (*Z* = −0.11). In addition, it should be noted that 69.1% of the students in cluster 1 were male.

Cluster 2: The second profile was named “Low Disruptive Behaviours Profile”. The profile comprised the vast majority of students (661; 87.2%). In this case, the highest *Z* score corresponded to intrinsic motivation (*Z* = 0.35), followed by extrinsic motivation, but with lower values that were closer to zero. The other factors scored negatively in this profile. As for the students who made up this cluster, unlike the other, the majority of students were female (57.6%).

### 3.3. Clusters’ Differences According to Sex and Age

An analysis of variance was conducted to check the differences between clusters according to sex and age of the participants. Differences in the interaction of sex × age (*F* = 0.31, *p* = 0.91, Cohen’s d = 0.00; power observed = 0.13) were not found. According to sex (*F* = 13.96, *p* < 0.0001, Cohen’s d = 0.04; power observed = 0.99), significant statistical differences were found, but no significant statistical differences according to exact ages of the students (*F* = 0.931, *p* = 0.477, Cohen’s d = 0.01; power observed = 0.33) were found (R^2^ = 0.12). The analysis of the residues can be seen in Table 4. Cluster 1 (high disruptive behaviours profile) was positively associated with boys. Meanwhile, cluster 2 (low disruptive behaviours profile) was positively associated with girls (Table 4).

### 3.4. Differences in School Satisfaction and Teaching Competence

Finally, to analyse the interaction of the clusters with school satisfaction and teaching competence, a multivariate analysis of variance (MANOVA) was carried out in which the clusters acted as an independent variable, and the ISC and ETCS-EF subscales acted as dependent variables. The homogeneity of covariance was examined using Box’s *M* test, and the null hypothesis of the data adjustment was rejected (Box’s *M* = 72.31, *F* = 11.91, *p* < 0.001). The suggestions of Tabachnick and Fidell [52] were followed, in which Pillai’s Trace was used instead of Wilk’s Lamda to assess the significance of the main effects and interactions. The multivariate contrast demonstrated significant differences and multivariate interaction effects (Pillai’s Trace = 0.46, *F*_(3, 755)_ = 12.03, *p* < 0.001, partial eta square = 0.05, observed power = 1.00). Significant differences were found in boredom with school, with higher mean values in cluster 1 of the high disruptive behaviours profile, and in the Evaluation of Teaching Competencies Scale, although in this case, the higher mean values corresponded to cluster 2 of the low disruptive behaviours profile (Table 5).

## 4. Discussion and Conclusions

The objective of the work was to analyse the disruptive behaviour and motivation profiles of secondary school students in PE classes as well as to study the differences, according to these profiles, regarding their satisfaction with school and teacher competence. The results provided two profiles. The first and least numerous, called the “High Disruptive Behaviours Profile”, encompassed a set of mostly boys with the lowest values being for intrinsic motivation and the highest for amotivation. It affirmed that they disrupted the class environment, disobeyed the rules, were irresponsible, and had low engagement in learning. These results were in line with those provided by Baños et al. [37] with students at the same educational level, which reflected that the boys obtained the highest means in behaviours related to irresponsibility and low engagement, disobeying the rules, disrupting the class environment, and having low personal self-management. In summary, Granero-Gallegos and Baena-Extremera [14] noted that the factors with the greatest negative prediction towards school satisfaction were irresponsibility and low engagement; thus, students who were irresponsible and had low engagement in academic tasks presented a low level of school satisfaction. Conversely, disrupting the classroom environment was established as a positive predictor of satisfaction/fun with school, possibly because this behaviour often occurs with students who try to make their classmates laugh, which gives them some personal satisfaction.

The second cluster was named the “Low Disruptive Behaviours Profile”. This profile characterized most of the students analysed and was composed mainly of girls, with the highest values being for intrinsic motivation, while scoring negatively in all the disruptive behaviours. With regard to this, Baños, Ortiz-Camacho, Baena-Extremera, and Zamarripa [53], and Baños et al. [37] showed that the girls obtained higher mean values in aggressiveness, contrary to what Cothran and Kulinna [13] had previously found in boys, while the behaviours of irresponsibility, low engagement, and disrupting the class environment were higher in girls. In this regard, Kulinna, Cothran, and Regualos [54] stated that it was the female students and female teachers who reported perceived worse behaviours in the PE class rather than the males. This may be because females tend to be more the victims of disruptive behaviours, and therefore report a higher incidence level [13].

Regarding the interaction of the two clusters with school satisfaction and teaching competence, the results showed significant differences for boredom with school, with higher mean values in the “High profile disruptive behaviours” cluster and on the Evaluation of Teaching Competencies Scale; although in this case, the highest mean values corresponded to the “Low Disruptive Behaviour Profile” cluster. Therefore, it was the students with the worst behaviours who claimed to be bored the most, with all that goes with it—potentially harming student learning, feeling excluded, and even dropping out of school.

The results showed that teaching competence was an important aspect for students in both clusters, although it was those students who exhibited the best behaviours in PE classes who perceived the greatest competence in their teachers. In the findings of Baena-Extremera et al. [36], it was shown that the greater the teacher’s competence, the greater the satisfaction/fun and the less the boredom felt amongst the students. These results are in line with those recently found by Baños et al. [37], which made clear that the lack of PE teacher competence, as perceived by adolescents, was positively related to the worst behaviours in PE classes, especially irresponsibility and low engagement, disobeying the rules, disrupting the class environment, and low personal self-management. The latter study also found that PE teacher competencies were significantly and directly related to aggressiveness. These authors, supported by Buscá, Ruiz, and Rekalde [55], noted that the results might be due to the eminently practical nature of PE and the interaction that occurs among students who are striving to win and to demonstrate adolescent skills in front of their peers. Accordingly, the excessive pursuit of victory through competition can lead to conflicts in PE classes [56]. As Macazaga, Rekalde, and Vizcarra [57] stated, it is much more common that conflicts arise in PE classes than in other subjects, where students sit still while performing tasks. To avoid these types of conflicts, it is important that the PE teacher acquires the maximum motivational skills and strategies appropriate to the specific characteristics and needs of his/her students, thus maintaining discipline in the classroom [31,58].

Different authors have provided various suggestions and guidelines to follow with regard to avoiding disruptive behaviours in PE classes and improving student learning. For example, according to Catano and Harvey [47], teachers should stand out for their good communication, creativity, work and social awareness, problem-solving skills, and professionalism. Kuzmanovic, Savic, Popovic, and Martic [59], on the other hand, highlighted the importance of the teacher being available to solve behavioural problems in the class, while Rasmussen, Scrabis-Fletcher, and Silverman [60] suggested that teachers should provide students with high-quality instruction, a variety of assignments, and individualized practice in order to improve these indiscipline issues.

Finally, responding to the hypothesis, it should be pointed out that students with a high disruptive behaviours profile were characterized as being mostly boys, with low levels of intrinsic motivation and high levels of amotivation and disruptive misbehaviour (disrupting the class environment, disobeying the rules, irresponsibility, and having low engagement with learning). In contrast, students with a low disruptive behaviours profile were usually girls, possessing the highest levels of intrinsic motivation and the lowest in all the disruptive behaviours. Lastly, the results show that the students with the worst behaviours were those who claimed to get bored the most in school, while those with better behaviours in PE classes perceived greater competence in their teachers.

This research highlights the importance of studying differences in relation to disruptive behaviour profiles and motivation among students, according to teacher competence and student satisfaction with school. Based on this research, recommendations can be made, both to the classroom and to school. Overall, we can recommend the creation or strengthening of classrooms for school coexistence, which contributes to reflection and improvement of commitment on the part of disturbing students, without punishment or sanctions, and to the resolution of conflicts in a positive and operative manner. By law, all schools must have a School Coexistence Plan, which must be implemented. Special emphasis should be placed on the development and improvement of this coexistence plan by the educational centers. More particularly, it is possible to focus on approaches that imply an enhancement of the intrinsic motivation among students, as well as the improvement of teaching competence in various aspects (e.g., communication, work awareness, availability, creativity, feedback, individual consideration of the student, problem-solving, social awareness, etc.), although the teacher should enhance their professional training in conflict resolution in the classroom. Related to this aspect, the educational administration must provide teachers with the necessary continuous training to improve social skills and face the challenges posed by disruptive behaviour and coexistence in today’s classrooms.

Regarding limitations of this research, it is necessary to take into account that the study was quantitative in that the sample was not representative; therefore, the results cannot be generalized, and the method used does not allow one to go deeper into the disruptive causes in the classroom. Furthermore, the high schools in which the data were collected by means of a questionnaire were not randomized. For future studies, mixed quantitative and qualitative research designs could be proposed, focusing on all subjects, not just PE. These quantitative designs would be developed with representative samples to generalize the results, with differences analysis according to sex and exact age, and qualitative designs that allow us to go deeper into the causes of disruptive behaviour. Some of these studies could also include private schools and public schools located in areas of low socioeconomic level (compensatory education centers). On the other hand, it would also be convenient to carry out longitudinal studies, with several data collections, in which the effectiveness of coexistence programs is valued.

## Figures and Tables

**Table 1:** Descriptive analysis and correlation between variables.

	*M*	*SD*	2	3	4	5	6	7	8	9	10
1. Low engagement or irresponsibility	2.00	0.90	0.70 **	0.64 **	0.59 **	−0.25 **	−0.18 **	0.18 **	0.01	0.26 **	−0.21 **
2. Fails to follow directions	1.65	0.84	−	0.71 **	0.66 **	−0.19 **	−0.14 **	0.26 **	0.06	0.18 **	−0.15 **
3. Distracts or disturbs others	1.49	0.79	−	−	0.79 **	−0.10 **	−0.05	0.24 **	0.04	0.24 **	−0.12 **
4. Poor self-management	1.42	0.83	−	−	−	−0.08 *	−0.04	0.20 **	0.04	0.19 **	−0.10 **
5. Intrinsic motivation	4.94	1.36	−	−	−	−	0.82 **	0.17 **	0.30 **	−0.12 **	0.43 **
6. Extrinsic motivation	4.85	1.25	−	−	−	−	−	0.29 **	0.29 **	−0.16 **	0.39 **
7. Amotivation	3.72	1.57	−	−	−	−	−	−	0.16 **	0.11 **	0.06
8. Satisfaction with school	2.80	0.87	−	−	−	−	−	−	−	−0.32 **	0.21 **
9. Boredom with school	3.03	1.04	−	−	−	−	−	−	−	−	−0.14 **
10. Teaching competence	5.36	1.16	−	−	−	−	−	−	−	−	−

Note: * *p* < 0.05; ** *p* < 0.01. *M* = mean; *SD* = standard deviation.

**Table 2:** Clusters of group A and group B following hierarchical method (Ward) and nonhierarchical method (k-means).

Subescales	Group A Ward’s Method (Hierarchical)				Group B k-Means Method (Nonhierarchical)					
	Cluster 1 (*n* = 43)		Cluster 2 (*n* = 328)		Cluster 1 (*n =* 50)			Cluster 2 (*n =* 336)		
	“High Disruptive Behaviors”		“Low Disruptive Behaviors”		“High Disruptive Behaviors”			“Low Disruptive Behaviors”		
	*M*	*SD*	*M*	*SD*	*M*	*SD*	*Z*	*M*	*SD*	*Z*
Low engagement or irresponsibility	3.48	0.92	1.82	0.69	3.56	0.68	1.73	1.75	0.67	−0.27
Fails to follow directions	3.33	0.93	1.46	0.56	3.12	0.87	1.75	1.41	0.52	−0.29
Distracts or disturbs others	3.23	0.94	1.29	0.40	3.07	0.93	2.00	1.24	0.38	−0.32
Poor self-management	3.26	0.98	1.22	0.46	3.07	1.05	1.98	1.14	0.31	−0.34
Intrinsic motivation	4.88	1.15	4.92	1.36	4.45	1.21	−0.36	5.08	1.37	0.20
Extrinsic motivation	4.80	1.08	5.02	1.31	4.59	1.10	−0.21	4.92	1.22	0.06
Amotivation	5.08	1.00	3.63	1.57	4.82	1.36	0.98	3.55	1.56	−0.14

Note: Sample (*n*), mean (*M*), standard deviation (*SD*), and *Z* values in the clusters according to the distribution of group A (*n* = 372; 49.1%) and group B (*n* = 386; 50.9%).

**Table 3:** Mean, standard deviation, and *Z* values in the clusters with the total sample.

Subescales	Cluster 1 (*n =* 97) “High Disruptive Behaviors”			Cluster 2 (*n =* 661) “Low Disruptive Behaviors”		
	*M*	*SD*	*Z*	*M*	*SD*	*Z*
Low engagement or irresponsibility	3.49	0.80	1.66	1.79	0.68	−0.24
Fails to follow directions	3.22	0.89	1.87	1.43	0.54	−0.26
Distracts or disturbs others	3.15	0.92	2.10	1.26	0.38	−0.30
Poor self-management	3.22	1.00	2.17	1.17	0.35	−0.31
Intrinsic motivation	4.59	1.31	−0.46	4.99	1.35	0.35
Extrinsic motivation	4.72	1.21	−0.11	4.87	1.25	0.13
Amotivation	4.59	1.36	1.15	3.60	1.56	−0.18

Note: *n* = sample; *M* = mean; *SD* = standard deviation; *Z* = standardized values.

**Table 4:** Clusters’ characteristics according to sex and age.

Subescales		Sex		Age					
		Boys	Girls	13	14	15	16	17	18
		*n* = 347	*n* = 411	*n* = 144	*n* = 232	*n* = 164	*n* = 153	*n* = 41	*n* = 24
		45.8%	54.2%	19.0%	30.6%	21.6%	20.2%	5.4%	3.2%
Cluster 1	*n* = 97% Residues	67	30	14	30	25	15	5	5
		69.10%	30.90%	14.40%	32.90%	26.80%	15.50%	5.20%	5.20%
		1.49	−1.49	0.92	0.83	0.76	−0.45	−0.85	−1.21
Cluster 2	*n* = 661% Residues	280	381	128	201	140	137	36	19
		42.4%	57.6%	19.4%	30.3%	21.1%	20.8%	5.5%	2.9%
		−0.52	0.52	−0.39	−0.60	−0.55	0.56	0.37	0.63

Note: *n* = sample; % = percentage.

**Table 5:** Differences in satisfaction with school and teacher competence according to cluster; multivariate analysis.

Subescales	Cluster 1		Cluster 2					
	*M*	*SD*	*M*	*SD*	*F*	*p*	*d*	Power Observed
Satisfaction with school	2.91	1.06	2.78	0.84	1.81	0.178	0.00	0.27
Boredom with school	3.46	1.04	2.97	1.02	18.92	0.000	0.41	0.99
Teaching competence	5.04	1.21	5.41	1.16	8.53	0.004	0.29	0.83

Note: *M* = mean; *SD* = standard deviation; *d* = Cohen’s d.
